# Implantable system for chronotherapy

**DOI:** 10.1126/sciadv.abj4624

**Published:** 2021-11-26

**Authors:** Seung Ho Lee, Qianqian Wan, Adam Wentworth, Ian Ballinger, Keiko Ishida, Joy E. Collins, Siddartha Tamang, Hen-Wei Huang, Canchen Li, Kaitlyn Hess, Aaron Lopes, Ameya R. Kirtane, Jung Seung Lee, SeJun Lee, Wei Chen, Kaitlyn Wong, George Selsing, Hyunjoon Kim, Stephen T. Buckley, Alison Hayward, Robert Langer, Giovanni Traverso

**Affiliations:** 1 The David H. Koch Institute for Integrative Cancer Research, Massachusetts Institute of Technology, Cambridge, MA 02139, USA.; 2 Division of Gastroenterology, Brigham and Women’s Hospital, Harvard Medical School, Boston, MA, 02115, USA.; 3 Department of Intelligent Precision Healthcare Convergence, Sungkyunkwan University (SKKU), Suwon 16419, Republic of Korea.; 4 Division of Comparative Medicine, Massachusetts Institute of Technology, Cambridge, MA 02139, USA.; 5 Global Research Technologies, Novo Nordisk A/S, Måløv, Denmark.; 6 Department of Chemical Engineering, Massachusetts Institute of Technology, Cambridge, MA 02139, USA.; 7 Department of Chemical Engineering, Massachusetts Institute of Technology, Cambridge, MA 02139, USA.

## Abstract

Diurnal variation in enzymes, hormones, and other biological mediators has long been recognized in mammalian physiology. Developments in pharmacobiology over the past few decades have shown that timing drug delivery can enhance drug efficacy. Here, we report the development of a battery-free, refillable, subcutaneous, and trocar-compatible implantable system that facilitates chronotherapy by enabling tight control over the timing of drug administration in response to external mechanical actuation. The external wearable system is coupled to a mobile app to facilitate control over dosing time. Using this system, we show the efficacy of bromocriptine on glycemic control in a diabetic rat model. We also demonstrate that antihypertensives can be delivered through this device, which could have clinical applications given the recognized diurnal variation of hypertension-related complications. We anticipate that implants capable of chronotherapy will have a substantial impact on our capacity to enhance treatment effectiveness for a broad range of chronic conditions.

## Teaser

A mobile app–controlled implantable system enables chronotherapy by controlling the timing of drug administration.

## INTRODUCTION

The timing of drug administration is important for treating a broad range of diseases. For example, the statin class of lipid-lowering drugs works best when taken before bedtime, because the level of its target enzyme, 3-hydroxy-3-methylglutaryl coenzyme A (HMG-CoA) reductase, peaks at night (*1*, *2*). Similarly, many studies have shown that taking antihypertensive agents is most effective at bedtime, which is related to the fact that the angiotensin 2 receptor, involved in blood pressure regulation, is maximally expressed at nighttime (*2*). The symptoms of some illnesses peak in diurnal patterns. For example, the most severe symptoms of asthma occur around 4 a.m. (*3*) and myocardial infarction and angina most commonly occur in the early morning hours (*4*). In addition, the circadian clock affects hormonal homeostasis with several hormones exhibiting daily oscillations. During daytime and feeding, insulin is released to ensure uptake of glucose, lipids, and amino acids (*5*), while growth hormone is released during nighttime (*6*) to promote fatty acid oxidation and insulin-like growth factor 1 release. Therapeutically, the ability to mirror these physiological circadian patterns can help mitigate adverse effects on metabolism such as increases in body weight, fatty liver development, and insulin resistance (*7*). Although the importance of relating the timing of drug administration has been revealed, it is still underappreciated in current medical practice (*8*).

Chronotherapy is the strategy of optimizing drug effectiveness by understanding the impact of delivery time. Taking medications at a specific time of day may also reduce toxic side effects (*9*). Chronotherapy takes into account the circadian clock, which drives the biological rhythm of behavior and physiology with a periodicity of approximately 24 hours (*10*). A phenomenon that has recently gained notable attention with the 2017 Nobel Prize in Physiology or Medicine awarded for discoveries of underlying cellular mechanisms that control circadian rhythms (*11*).

In the practice of clinical medicine, chronotherapy has great potential to markedly improve treatment outcomes, but delivering drugs at specific and consistent times of the day remains a considerable challenge. To begin with, it is difficult for patients to adhere to a prescribed schedule; only about 50% of individuals with chronic diseases adhere to their treatment regimen(s), according to the World Health Organization (*12*). Taking the medication at a specified time adds an additional level of adherence complexity. Second, extended-release systems, such as implantable devices (*13*, *14*), nano-/microparticles (*15*), hydrogels (*16*, *17*), and microneedle patch systems (*18*, *19*), can improve medication adherence with the exception that surgically implanted systems cannot deliver drugs at a time during the drug delivery interval. These delivery systems mostly allow diffusion-based continuous drug release, a strategy that cannot be applied to chronotherapy. Examples such as the etonogestrel implant Nexplanon and the exenatide implantable mini-pump ITCA650 only allow for a predetermined, continuous drug release (*13*, *14*, *20*). Similarly, while engineering the pharmacokinetic properties of biologics, e.g., via fatty acid acylation, PEGylation, Fc-fusion, etc., confers extended half-life and thereby enhanced convenience and therapeutic efficacy, aspects relating to circadian activity are not addressed by these approaches.

We hypothesized that therapeutic effectiveness could be optimized by a drug delivery approach that leverages chronotherapy to deliver drugs with precision timing while decreasing the adherence burden on the patient by providing extended drug delivery. To address this, we designed an implantable pump capable of extended drug delivery that can release discrete doses at specific times. Our wirelessly controlled battery-free implantable system (WCBIS) is designed to increase the likelihood of patient acceptability. Most active implantable drug delivery pumps are somewhat bulky to accommodate the mechanical component and/or powered by an electronic circuit and battery (*21*, *22*), which occupy a minimum volume of 2.57 to 67.5 ml and require longer recovery periods and/or surgery for battery replacement (table S1). In contrast, our small, cylindrically shaped pump is battery-free to decrease the need for exchange and moreover is compatible with trocar-needle administration. Here, we show that it can deliver accurate amounts of drugs at a specific time when actuated wirelessly by the wearable controller.

## RESULTS

### Design and operated features of WCBIS

**Fig. 1.**
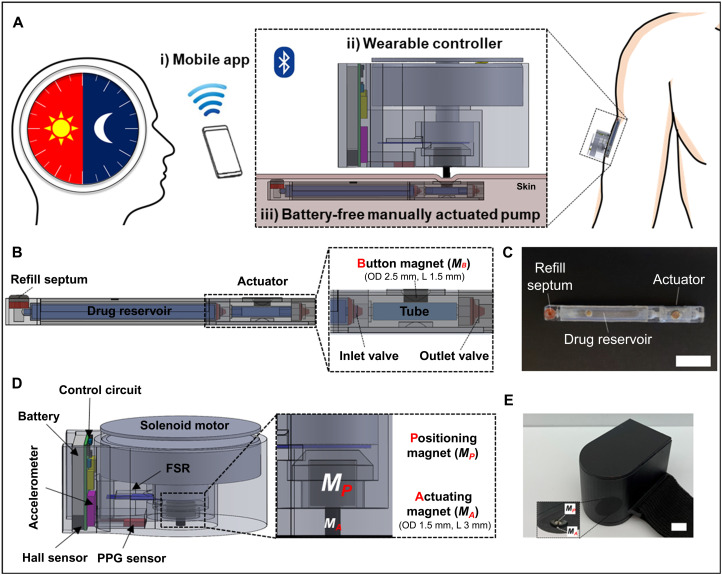
Descriptive illustrations and images of wirelessly controlled battery-free implantable system (WCBIS) for chronotherapy. (**A**) Three-dimensional schematic of the WCBIS. (**B** and **C**) CAD drawing and image of the battery-free manually actuated pump (scale bar, 10 mm). (**D** and **E**) Computer-Aided Design (CAD) drawing and image of the wearable controller (scale bar, 10 mm). FSR, force sensitive resistor. Photo credit: Seungho Lee, Massachusetts Institute of Technology.

The system combines a battery-free, manually actuated pump, a wearable controller, and a mobile app (Fig. 1A). The small, cylindrically shaped pump consists of a drug reservoir with a refill septum and an actuator that is assembled with an inlet valve, a fluid chamber, a button magnet, and an outlet valve in series (Fig. 1B). The detailed assembly procedure is shown in fig. S1. Notably, the pump does not need a battery since there are no electronic circuits inside the device and thus can be designed with a small volume for minimally invasive implantation. The pump is similar in size to the abovementioned passive subdermal implants (*13*, *14*, *20*), but drug release can still be controlled at specific times from outside the body.

To prevent an accidental or unwanted actuation, the button magnet in the actuator is designed to be small so that the pump cannot be driven even when pressed with a baby’s small finger. To extend the pump’s life span, it has a flexible drug reservoir with a high volumetric loading efficiency and a protruding septum for easy refilling via 30-G syringe needle injections after implantation (fig. S2). Figure 1C shows an image of the battery-free manually actuated pump. The pump prototype measures 4 mm in diameter and 46 mm in length. The wearable controller is designed to be located outside the skin and mainly consists of the solenoid motor, FSR (force sensitive resistor), Hall effect sensor, accelerometer, PPG (photoplethysmography), and control circuit, as shown in Fig. 1D. Figure 1E shows an optical image of the wearable controller; the total volume is approximately 31.3 ml, measuring 50 mm by 25 mm by 25 mm (*l* × *w* × *h*).

**Fig. 2.**
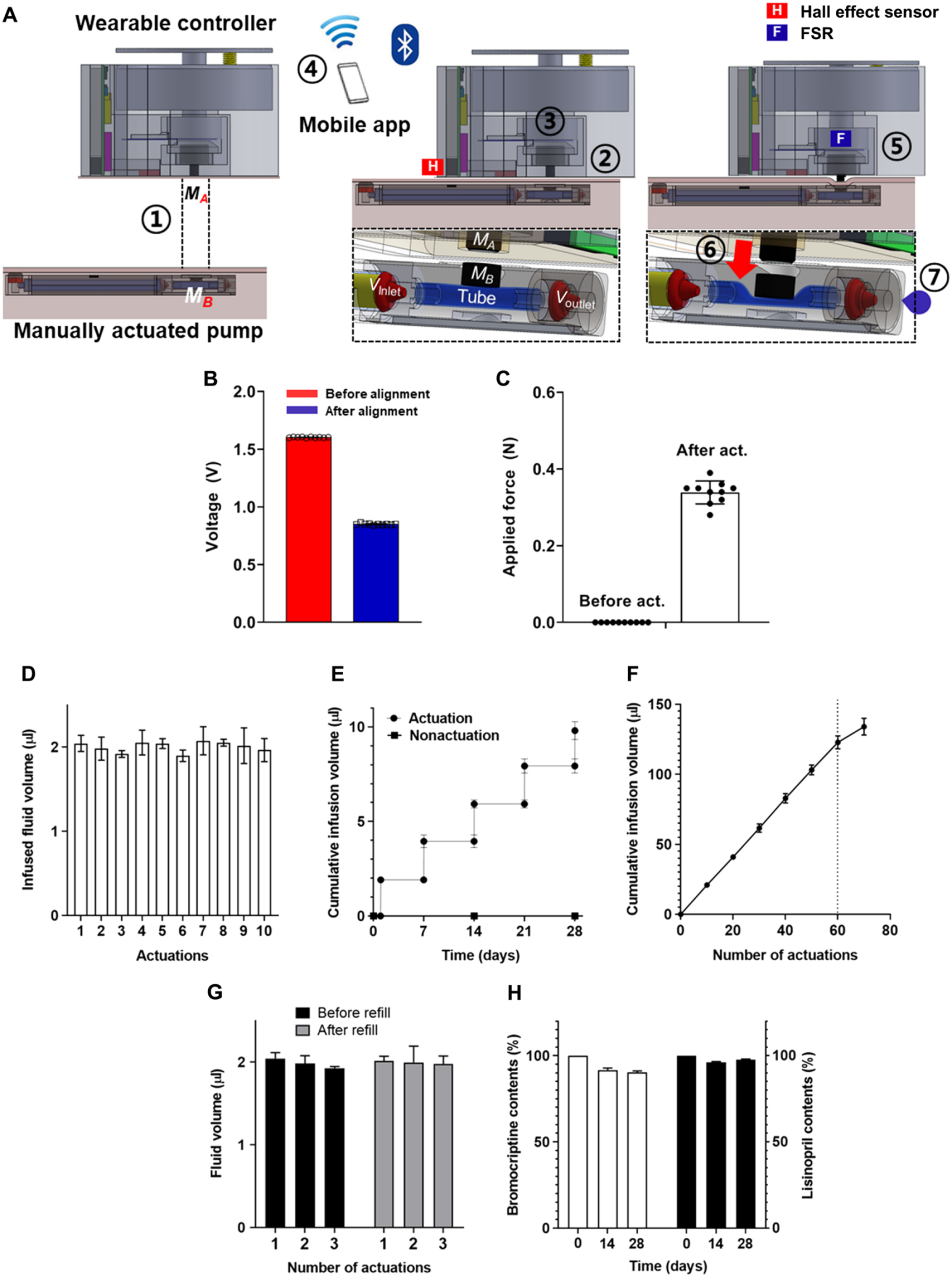
Electrical and fluidic characterization of WCBIS. (**A**) Actuation principle of the WCBIS. First, the wearable controller can be easily attached over the implanted pump due to the opposite polarities between a positioning magnet and button magnet (①). Then, proper alignment with the pump is confirmed using a Hall effect sensor, which is integrated at the bottom of the controller (② and B). When the drug infusion is needed, the controller operates the solenoid motor (③) using a mobile app via Bluetooth (④). Then, the actuating magnet moves downward (⑤), and the tube is compressed (⑥), releasing the drug (⑦). (**B**) Hall effect sensor output indicating alignment. (**C**) Applied force of actuator, as measured by FSR sensor. (**D**) Fluid volume infused from the pump per actuation. Ten consecutive actuations were applied with a wearable controller (*n* = 3). (**E**) The pumps were tested with and without actuation in saline at predetermined times on days 1, 7, 14, 21, and 28 (*n* = 3). (**F**) Accelerated depletion test. (**G**) Refill test. The pumps filled with lisinopril (10 mg ml^−1^) were tested three times before and after refill, respectively (*n* = 3). The infused fluid volume per actuation was highly reproducible, indicating that the pump’s performance was not affected by the refilling procedure. (**H**) Stability evaluation of bromocriptine and lisinopril was assessed with high-performance liquid chromatography (*n* = 3) after being stored in the pump at 37°C for 28 days. Data are shown as means ± standard error.

To enable programmable drug release, we developed an iOS-based mobile app using a graphical user interface (GUI) that can generate commands to control the wearable controller, as shown in fig. S3. Figure 2A shows the working principle of the WCBIS. Proper alignment between the controller and the pump is confirmed using a Hall effect sensor, which is integrated at the bottom of the controller (Fig. 2B). The FSR is mounted at the top of the positioning magnet; it receives feedback, indicating complete pump actuation, hence ensuring appropriate drug infusion (Fig. 2C).

### In vitro performance test

As shown in movie S1 and Fig. 2D, the bromocriptine had a jet-like infusion and the infused fluid volume per actuation was 2.0 ± 0.12 μl, indicating high reproducibility even with 10 consecutive actuations. To evaluate long-term feasibility, the pump was immersed in phosphate-buffered saline (PBS) for 28 days. After immersion, the average infused fluid volume per actuation was 1.96 ± 0.14 μl, similar to the preimmersion volumes, again showing high reproducibility; also, no drug was detected during nonactuation periods (Fig. 2E). The flexible drug reservoir (i.e., 150 μl) enabled the pump to infuse a reproducible volume per actuation of 1.93 ± 0.12 μl with up to 60 consecutive actuations until approximately 89.1% of the total solution volume in the drug reservoir was consumed (Fig. 2F). As shown in Fig. 2G, the drug infusion profile was not affected by the refilling procedure. Each bromocriptine and lisinopril formulation loaded into the device was stable without apparent degradation when stored at 37°C for 2 or 4 weeks (Fig. 2H).

### In vivo evaluation

To demonstrate the effect of chronotherapy, we evaluated the WCBIS for two different applications. One was for treating type 2 diabetes using bromocriptine, which is a sympatholytic D2 dopamine agonist and has inhibitory effects on serotonin turnover within the central nervous system (*23*). The other was for treating hypertension using a lisinopril, which is an angiotensin-converting enzyme (ACE) inhibitor (*24*).

**Fig. 3.**
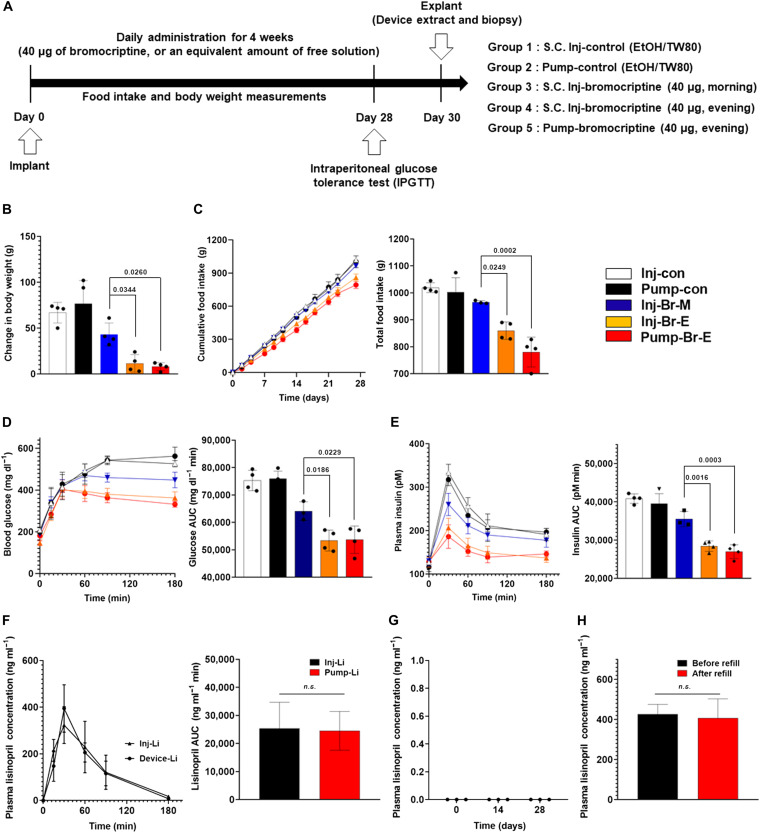
Therapeutic efficacy of the WCBIS in vivo models. (**A**) Therapy protocol used to investigate the pump in ZDF diabetic rat model. EtOH, ethanol. (**B** and **C**) Profiles of (B) body weight change, (C) cumulative food intake, (**D**) blood glucose levels, and (**E**) plasma insulin levels after the 4-week treatment of ZDF rats (*n* = 4). (**F**) In vivo lisinopril pharmacokinetic test. Profiles of plasma concentration of lisinopril measured from the blood sampled at 0, 15, 30, 60, 120, and 180 min after lisinopril administration. The maximum plasma concentration of lisinopril was measured at 30 min in both the pump (*n* = 3) and the subcutaneous injection groups. (**G**) Long-term leakage test. When there was no actuation, lisinopril was not detected with the measurement method at days 0, 14, and 28. (**H**) Reproducibility assessment of the pump after a refill in vivo. The pump loaded with lisinopril was implanted in SD rats (*n* = 3) and actuated before and after a refill procedure. After each actuation, the plasma concentration of lisinopril was measured at 30 min in both the prerefill and postrefill injection groups. The lisinopril concentration did not vary significantly, showing that the in vivo pump’s performance was not affected by the refilling procedure. All data are expressed as the means ± standard error. Statistical differences between the groups were determined with a one-way analysis of variance (ANOVA) followed by the Tukey’s post hoc test. All *P* values to determine statistical significance are presented in the graphs (C to E). Photo credit: Seungho Lee, Massachusetts Institute of Technology.

First, we investigated the in vivo pharmacodynamics (PD) of bromocriptine using Zucker diabetic fatty (ZDF) rats, a well-accepted type 2 diabetes model. The animals, separated into five groups, were given daily administrations of 40 μg for 4 weeks according to the following conditions: subcutaneous injection of a bromocriptine-free solution (Inj-con), pump infusion of a bromocriptine-free solution (Pump-con), morning subcutaneous injection of bromocriptine (Inj-Br-M), evening subcutaneous injection of bromocriptine (Inj-Br-E), and evening pump infusion of bromocriptine (Pump-Br-E) (Fig. 3A). To evaluate the pharmacologic effects in each group, we measured the animals’ body weights and food intake (*25*, *26*). The body weight gain and the total food intake in all three bromocriptine groups were lower than in both control groups (Fig. 3, B and C). The animal groups that received bromocriptine in the evening (Inj-Br-E and Pump-Br-E) showed a significantly lower body weight gain and total food intake than those treated by injection in the morning (Inj-Br-M) (*P* < 0.05).

During the intraperitoneal glucose tolerance test (IPGTT), which was conducted after 4 weeks of treatment, all three bromocriptine groups performed better than the control groups with respect to postprandial glucose metabolism. The postload glucose levels and the plasma glucose area under the curve (AUC_glucose_) for both evening bromocriptine groups (Inj-Br-E and Pump-Br-E) were significantly lower than those of the morning bromocriptine group (Inj-Br-M) (Fig. 3D). The plasma insulin concentrations during the IPGTT and AUC_insulin_ for both evening bromocriptine groups were comparably lower than those in the morning bromocriptine group (Fig. 3E). In addition, there was no statistically significant difference between the evening injection (Inj-Br-E) and evening pump (Pump-Br-E) groups, indicating that the bromocriptine efficacy delivered from the pump was comparable to that administered by subcutaneous injections. Although bromocriptine’s mechanism of action is not fully understood, the present results are consistent with data from many vertebrate species, showing that timed bromocriptine administration improves glucose tolerance (*27*).

To demonstrate the feasibility of chronotherapy of hypertension, we evaluated the pharmacokinetic profiles of lisinopril with the pump and the subcutaneous injection groups. As shown in Fig. 3F, the peak plasma concentrations of lisinopril (*C*_max_) for both the pump and injection groups were similar: 394.2 ± 83.2 ng ml^−1^ and 322.7 ± 64.7 ng ml^−1^, respectively. The time of maximum concentration observed (*T*_max_) for both groups was 30 min. Here, the AUC for plasma lisinopril concentration for both the pump and subcutaneous injection groups was 25,335.5 ± 7640.9 ng ml^−1^ min and 24,484.5 ± 5643.8 ng ml^−1^ min, respectively, which were not significantly different (*P* > 0.05). Over a 28-day period, there was no drug leakage detected from the implanted pump during the nonactuation periods (Fig. 3G). Also, the plasma concentrations of lisinopril at 30 min, C_max_, were not significantly different, indicating that the in vivo pump’s performance was not affected by the refilling procedure (Fig. 3H).

**Fig. 4.**
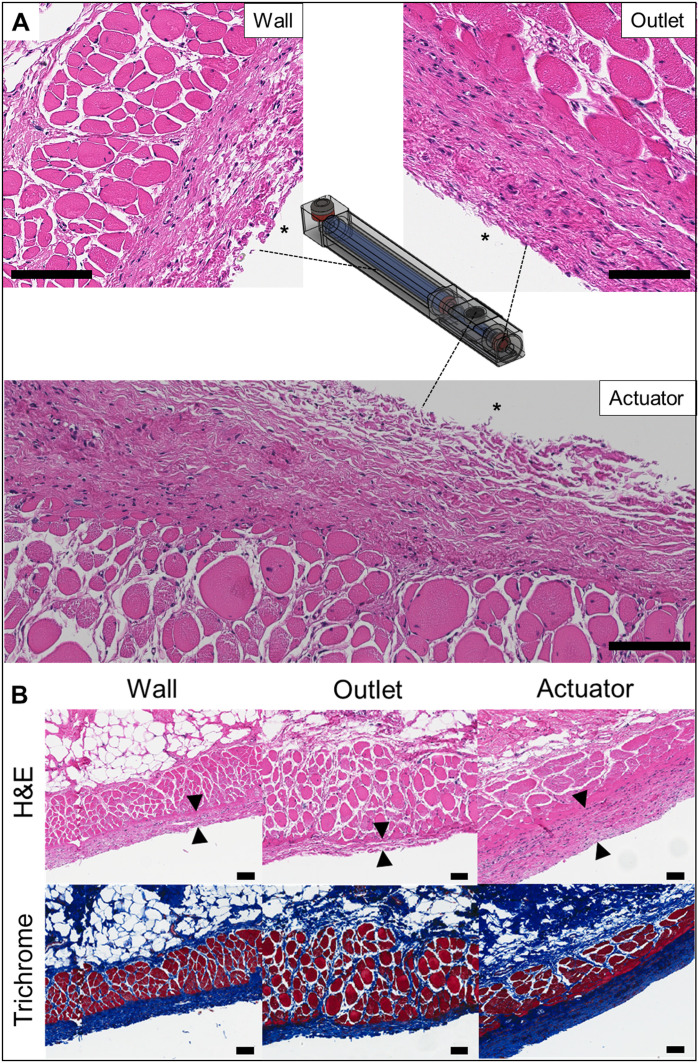
Representative histological images surrounding the implanted device. (**A**) Three distinct tissue locations surrounding the device and H&E-stained histological images for the assessment of inflammation. The asterisk (*) indicates the location of the implanted pump (*n* = 4). Scale bars, 100 μm. (**B**) H&E- and MT-stained images for the assessment of fibrous capsule formation. The arrows show the fibrous capsule formed surrounding the implanted device (*n* = 3). Scale bars, 100 μm. Photo credit: Seungho Lee, Massachusetts Institute of technology.

To evaluate the in vivo biocompatibility of the pump, three biopsied tissue samples were examined by both hematoxylin and eosin (H&E) and Masson’s trichrome (MT) staining from the following locations: (i) the outlet, (ii) the area for actuation (i.e., the actuator), and (iii) the wall. As shown in Fig. 4A, the overall inflammatory response was observed to be minor for all tissue locations. The tissue over the actuation area did not exhibit any signs of irritation after repetitive actuations. The MT-stained tissues showed fibrous capsule formation around the implanted device; the capsule thickness was 140.7 ± 30.01 μm (Fig. 4B). Notwithstanding, at the end of the experiments, the outlet in the pump was not blocked (i.e., 30 days after implantation).

## DISCUSSION

Chronotherapy is the timing of drug delivery that considers the patient’s circadian rhythm to attain optimal efficacy. Although it can effectively improve drug efficacy and reduce toxic side effects, taking a medication at a specified time contributes to an additional level of medication adherence complexity. In particular, most current extended-release systems that allow only diffusion-based continuous drug release are incompatible with chronotherapy (*13*–*20*).

Therefore, an active implantable device capable of on-demand/programmable delivery of a therapeutic agent at a specified time holds great promise for chronotherapeutic approaches. However, many such devices require electronic circuit components and batteries, which make them relatively large for implantation; they also require additional surgery for battery replacement (*21*, *22*). Since patient acceptance is the key to the success of implantable systems, the complexity and limitations of surgical procedures for implantation and explanation can have a considerable impact on the device acceptance in patients (*28*).

To address these challenges, in this work, we developed a WCBIS capable of an on-demand/programmable drug delivery by enabling the synchronization of drug administration with the time of delivery. Previously, several battery-free implantable devices have been proposed, for example, a soft implantable drug delivery device using a wireless power transmission (*29*) and, in another instance, a wirelessly controlled, bioresorbable drug delivery device that combines biocompatible wireless power-harvesting function (*30*). These devices rely on electronics, do not have refill features, and require an incision for implantation, which can limit subject acceptability. In contrast, the WCBIS represents a mechanically driven, refillable system that is compatible with trocar implantation to help maximize translatability.

Notably, the WCBIS described here was designed to be small, subcutaneous, and trocar-compatible like passive subdermal implants such as Nexplanon and ITCA650 (*13*, *14*). These features would be beneficial for ease of implantation/explantation through local anesthesia, further enhancing patient acceptability. Briefly, our pump size is similar to that of passive subdermal implants that only allow diffusion-based continuous drug delivery, but our pump still enables on-demand/programmable drug delivery at a scheduled time (Fig. 2, D to G, and fig. S4).

After one-time implantation, the WCBIS can consistently infuse an accurate bolus of drugs only when required. The drug dose can also be easily controlled by changing the number of actuations and the concentration of drug solution in the reservoir. In addition, the pump is equipped with a refill septum, whereby a fresh drug solution can be successfully supplied via a 30-G needle while the pump is still implanted. This refill procedure did not affect the reproducibility of drug infusion, allowing semipermanent use after implantation (Fig. 2H). For the whole experimental period, we did not observe any rotation issue because the button magnet in the implanted pump always tends to attach to the positioning magnet in a wearable controller.

Here, this pump includes a safety feature to prevent an accidental or unexpected actuation. First, the button magnet in the actuator is designed to be smaller than a baby’s finger and is also designed to be properly operated under the pain threshold (fig. S5) (*31*). The force required when actuating the pump can be easily modified by changing the size of the actuating magnet. To approximate implantation in the upper arm (*32*), at each actuation, we used a 2-mm-thick SynTissue brand segment, as the intervening tissue, recognizing that it has mechanical characteristics approximating live human tissue (*33*) (Fig. 2, D to G). In rats, the pumps implanted were successfully operated in long-term actuations (i.e., daily for 28 days) without anesthesia (Fig. 3), and histopathology results showed that there was no adverse effect at any tissue locations (Fig. 4). In addition, the mobile app was programmed to preset restrictions for misalignment, improper actuations, and the drug infusion schedule to prevent overdosing (figs. S3 and S6).

In this study, our pump exhibited in vivo PD profiles of bromocriptine for type 2 diabetes in ZDF rats similar to profiles with the subcutaneous injection (Figs. 3 and 4). In addition, we also have demonstrated that lisinopril for hypertension can be successfully infused through this WCBIS, by attaining a subcutaneous injection-like pharmacokinetic profile.

For clinical applications, changes may need to be made to optimize safety and patient acceptability. For future versions of the system, it would be desirable to choose high-strength biomaterials, such as stainless steel or titanium (*34*). For implantation in humans, this system has a small fixed operating unit volume for actuation without complex control units and batteries. Thus, it can be easily optimized by changing only the drug reservoir volume, which can be adjusted according to the treatment period and the number of drug refills required (table S1).

In addition, the wearable controller could be miniaturized further to dimensions similar to those of existing U.S. Food and Drug Administration–approved blood glucose monitoring systems, such as Eversense and Dexcom, since these devices have already demonstrated their feasibility in clinical settings (table S3) (*35*, *36*). Also, incorporating sensors in a wearable controller that can track and record the daily rhythms in sleep could make a chronotherapy approach more feasible (fig. S7) (*37*, *38*). Further studies exploring a range of drugs with short half-lives are warranted since the clinical translation of timed drug delivery using the wirelessly controlled implantable system could transform the clinical landscape of therapeutics in chronotherapy (*38*). In conclusion, we have proposed the WCBIS as an attractive device platform for chronotherapy that affords a number of advantages: (i) It is a small-scale and compact implant, which could increase patient acceptability; (ii) it allows on-demand/programmable delivery of a therapeutic agent at specified times; and (iii) it enables semipermanent use (i.e., battery-free and refillable) without replacement surgery. High dosing accuracy per actuation of the pump is maintained until most of the drug within the flexible drug reservoir is consumed, resulting in less frequent refills. Notably, the WCBIS demonstrates the pharmacokinetic profiles and pharmacodynamic effects similar to that of subcutaneous injection at a specified time mimicking the circadian rhythm. We conclude that the proposed WCBIS can be a promising treatment tool with high patient acceptability that can deliver drugs at specific times for personalized chronomedicine and circadian health.

## MATERIALS AND METHODS

### HPLC-UV analysis

To evaluate the pump performance under in vitro environments, the concentration of bromocriptine mesylate (MedchemExpress, NJ, USA) and lisinopril (Sigma-Aldrich, MO, USA) in PBS (Gibco) was measured by high-performance liquid chromatography (HPLC) with an Eclipse XDB C18 column (5 μm, 4.6 mm by 150 mm; Agilent Technology) and a Zorbax Eclipse XDB C18 column (4.6 mm by 150 mm, 5 μm; Agilent Technology), respectively.

In the case of bromocriptine, the optimized mobile phase consisted of 10 mM ammonium acetate at a pH of 4.00 in water and methanol (30:70, v v^−1^) at a flow rate of 1.5 ml min^−1^ over a 10 min run time. The injection volume was 10 μl, and the bromocriptine concentration was measured at 240 nm.

For lisinopril, the mobile phase consisted of a mixture of 20 mM dipotassium phosphate at a pH of 3.00 in water and acetonitrile (55:45, v v^−1^) and was forced at 1 ml min^−1^ for an HPLC run time of 5 min. For each analysis, a 5-μl sample was injected into the column, and ultraviolet (UV) absorbance was monitored at λ_max_ = 210 nm.

### UPLC-MS/MS analysis

Analyte concentrations in plasma from in vivo experiments were analyzed using ultra-performance liquid chromatography (UPLC)–tandem mass spectrometry. Analysis was performed on a Waters ACQUITY UPLC-I-Class System aligned with a Waters Xevo TQ-S mass spectrometer (Waters Corporation, Milford MA). Liquid chromatographic separation was performed on an Acquity UPLC BEH C18 (50 mm × 2.1 mm, 1.7-μm particle size) column for lisinopril at 50°C. The mobile phase consisted of aqueous 0.1% formic acid, 10 mM ammonium formate solution (Mobile Phase A), and acetonitrile: 10 mM ammonium formate, 0.1% formic acid solution (95:5 v v^−1^) (Mobile Phase B). The mobile phase had a continuous flow rate of 0.6 ml min^−1^ using a time and solvent gradient composition. The initial composition, 100% Mobile Phase A, was held for 0.50 min. Then, the composition was changed linearly to 50% Mobile Phase A and 50% Mobile Phase B until 1.00 min. At 1.50 min, the composition shifted to 20% Mobile Phase A and 100% Mobile Phase B at 2.50 min. The composition was held constant at 100% Mobile Phase B until 3.00 min. At 3.25 min, the composition returned to 100% Mobile Phase A, where it remained at column equilibration for the duration of the run, ending at 4.00 min. A 12-point calibration curve was prepared from analyte-free, blank rat serum ranging from 1.25 to 5000 ng ml^−1^. Forty microliters of each serum sample was spiked with 80 μl of 250 ng ml^−1^ internal standard in acetonitrile to elicit protein precipitation. Then, 10 μl was injected for lisinopril.

Samples were introduced and ionized by electrospray ionization (ESI) in the positive ionization mode. Waters MassLynx 4.1 software was used for data acquisition and analysis. The mass-to-charge transitions (*m/z*) used to quantitate lisinopril were 406.39 > 84.20 and 406.39 > 246.27 for quantitation and confirmation, respectively. For the internal standard, enalaprilat, 349.33 > 206.30 and 349.33 > 117.16 *m/z* transitions were used for quantitation and confirmation, respectively.

### In vitro performance test

Here, bromocriptine formulations have been screened with different solvents and lastly selected with ethanol [(EtOH)/Tween 80 (1:1 (v v^−1^)] (table S2). Then, the pumps filled with 150 μl of bromocriptine solution (20 mg ml^−1^) were immersed in PBS (pH 7.4) at 37°C and were actuated using the wearable controller for 10 consecutive times.

The drug release profiles were determined by measuring the drug concentrations after actuating the device on days 1, 7, 14, 21, and 28. In addition, for the leakage test, devices were fully immersed in PBS for 4 weeks without any actuation. A safety test was also performed by the wearable controller via the mobile app. All experiments were tested three times for each scheduled time point, and the collected aliquot was analyzed using an HPLC-UV mentioned above.

### Accelerated depletion test

The pumps filled with 10 mg ml^−1^ of lisinopril solution were immersed in saline and consecutively actuated over 60 times until depletion. Then, the aliquot collected right after the operation of the pump was measured by the HPLC, and the cumulative amount of lisinopril release was obtained. To prepare the lisinopril solution, lisinopril in powder form was dissolved in sterile PBS at pH 7.4.

### Assessing drug stability

Bromocriptine (20 mg ml^−1^) and lisinopril (50 mg ml^−1^) solution were filled in the drug reservoir and stored at 37°C for 14 and 28 days. Then, the drug stability was tested by comparing a fresh solution of bromocriptine and lisinopril (day 0). Bromocriptine and lisinopril concentrations were assessed with HPLC, as described in Materials and Methods. After incubating for 28 days, the bromocriptine and lisinopril concentrations, as well as the retention time, did not change significantly.

### Drug refillability

To investigate the effect of drug refilling on the drug release profile, pumps filled with lisinopril (10 mg ml^−1^) were immersed in PBS (pH 7.4) and then compared before and after the refilling procedure. The in vivo refilling procedure was also performed when the pump was implanted in Sprague Dawley (SD) rats. Here, the protruding refill septum allows ease of detection on the skin above the implanted pump. Through this refill septum, we removed the remaining drug solution from the drug reservoir and fresh drug solution was supplied via a 30-G needle while the pump was still implanted (fig. S2). The maximum plasma concentration of lisinopril measured at 30 min was not significantly different, showing that in vivo performance of the pump was not affected by the refilling procedure.

### In vivo experiments

The aim of this study was to evaluate the efficacy and safety of the WCBIS for chronotherapy. For in vivo evaluation for type 2 diabetes, we used 12-week-old, male ZDF (fa/fa) rats (350 to 400 g; Charles River Laboratories, Wilmington, MA, USA).

The animals with blood glucose level > 300 mg/dl were divided into five groups: (i) Inj-con: animals treated with subcutaneous injection of EtOH/Tween 80 without bromocriptine; (ii) Pump-con: animals treated with the implanted pump filled with EtOH/Tween 80 without bromocriptine; (iii) Inj-Br-M [morning dosing (40 μg), 9:30 to 10 a.m.]: animals treated with subcutaneous injection of bromocriptine; (iv) Inj-Br-E [evening dosing (40 μg), 5:30 to 6 p.m.]: animals treated with subcutaneous injections of bromocriptine; and (v) Pump-Br-E groups [evening dosing (40 μg), 5:30 to 6 p.m.]: animals treated with the implanted pump loaded with bromocriptine.

For all groups, the same volume of bromocriptine or EtOH/Tween 80 [1:1 (v v^−1^)] without bromocriptine was administered daily for 4 weeks. For in vivo evaluation for hypertension, we filled the pump with lisinopril and used 12-week-old male SD rats (350 to 400 g; Charles River Laboratories, Wilmington, MA, USA) whose pharmacokinetic profiles were compared with the injection group.

For in vivo evaluation, the pumps were implanted subcutaneously in a rat model. The rats were anesthetized under isoflurane and received buprenorphine (0.05 mg kg^−1^ of body weight) before surgery. All animal protocols were approved by the Committee on Animal Care at the Massachusetts Institute of Technology. The animal room was on a 12-hour light/12-hour dark cycle. Humidity and temperature were kept at 40 to 60% and 19° to 23°C, respectively. A special diet (Purina 5008, Research diets, New Brunswick, NJ, USA) and water were provided ad libitum during the experiments for all ZDF rats.

### Intraperitoneal glucose tolerance test

The IPGTT was tested with all animal groups after 4 weeks of delivery with bromocriptine or controls. Animals were fasted for approximately 16 hours and had free access to water. Fasted blood glucose levels were determined before a glucose solution (2 g kg^−1^) was intraperitoneally administered. For blood glucose measurements, blood samples were obtained from the tail vein and were measured at 0, 15, 30, 60, 90, and 180 min using a glucometer (Accu-Check, Roche Diagnostics). A blood sample was taken at the same scheduled times and the insulin level was measured using a rat insulin ELISA (enzyme-linked immunosorbent assay) kit.

### Pharmacokinetics of lisinopril delivered from the pump

For the pharmacokinetic evaluation, 0.2 ml of blood was obtained at 0, 15, 30, 60, 90, and 180 min after lisinopril administration (20 μg). The collected samples were centrifuged at 5000 rpm for 15 min to separate the plasma and stored at −20°C. For the pharmacokinetic analysis, the area under the blood concentration versus time curve (AUC) was calculated by the trapezoidal rule.

### Histology

At the end point of the experiments, the ZDF rats were anesthetized with isoflurane, and a terminal cardiac blood collection was performed. Here, the tissues surrounding the device were biopsied from three distinct locations to investigate the biocompatibility of the implanted device, as depicted in Fig. 4. Each tissue sample was then prepared into slides, which were stained with MT and H&E to measure the fibrous capsule thickness around the implanted device and to assess the number of polymorphonuclear cells, respectively. The stained slides were analyzed using an optical microscope at ×200 magnification.

### Statistical analysis

One-way analysis of variance (ANOVA) followed by the Tukey’s post hoc test was used for multiple comparison. The repeated measure ANOVA was used to assess the effects of IPGTT. Statistical analysis was conducted using Prism 8.0 (GraphPad Software). The significance level was set at *P* < 0.05.

## Supplementary Materials

### This PDF file includes:

Figs. S1 to S7 Tables S1 to S3 Legend for movie S1 References

## Notes

View/request a protocol for this paper from *Bio-protocol*.
