# Supplementary material for: Implantable system for chronotherapy
Source: Sci Adv. 2021 Nov 26;7(48):eabj4624. doi: 10.1126/sciadv.abj4624 (PMC8626078; doi:10.1126/sciadv.abj4624)
Supplement: Supplementary file 1 — Figs. S1 to S7 Tables S1 to S3 Legend for movie S1 References [file sciadv.abj4624_sm.pdf]

Science Advances
AAAS

Supplementary Materials for

Implantable system for chronotherapy

Seung Ho Lee, Qianqian Wan, Adam Wentworth, Ian Ballinger, Keiko Ishida, Joy E. Collins, Siddartha Tamang, Hen-Wei Huang, Canchen Li, Kaitlyn Hess, Aaron Lopes, Ameya R. Kirtane, Jung Seung Lee, SeJun Lee, Wei Chen, Kaitlyn Wong, George Selsing, Hyunjoon Kim, Stephen T. Buckley, Alison Hayward, Robert Langer, Giovanni Traverso*

*Corresponding author. Email: ctraverso@bwh.harvard.edu, cgt20@mit.edu

Published 26 November 2021, *Sci. Adv.* **7**, eabj4624 (2021)
DOI: 10.1126/sciadv.abj4624

The PDF file includes:

Figs. S1 to S7
Tables S1 to S3
Legend for movie S1
References

Other Supplementary Material for this manuscript includes the following:

Movie S1

Table S1 Volume information of active implantable drug delivery pumps

Name	Total volume (ml)	Reservoir volume (ml)	Total-Reservoir volume (ml)
**WCBIS**	0.578	0.15	0.428
**iPRECIO pump** *(39)*	2.7	0.13	2.57
**Magnetic pump** *(40)*	7.82	0.6	7.22
**Microchip** *(21)*	18.4	0.07	18.33
**SynchroMed II pump (Medtronics)** *(41)*	87.5	20	67.5

Table S2 List of solvents used in formulation of bromocriptine

Solvent	Solubility (mg ml^{-1})
PBS (pH 7.4)	< 1
Sesame oil	< 5
corn oil	< 5
soybean oil	< 5
olive oil	< 5
peanut oil	< 5
Lard oil	< 5
Rapeseed oil	< 5
Cottonseed oil	< 5
Linseed oil	< 5
Sunflower seed oil	< 5
Ethanol	~ 7
Tween 80	~ 5
Ethanol :Tween80 (1:1, v v^{-1}) *(42)*	< 22

Table S3 Volume information of wearable devices

	Dimension (L x W x H, mm^3)	Total volume (ml)
**WCBIS**	50 x 25 x 25	31.3
**Eversense®** *(35)*	38 x 48 x 9	16.4
**Dexcom G6®** *(36)*	41 x 22 x 8	7.2

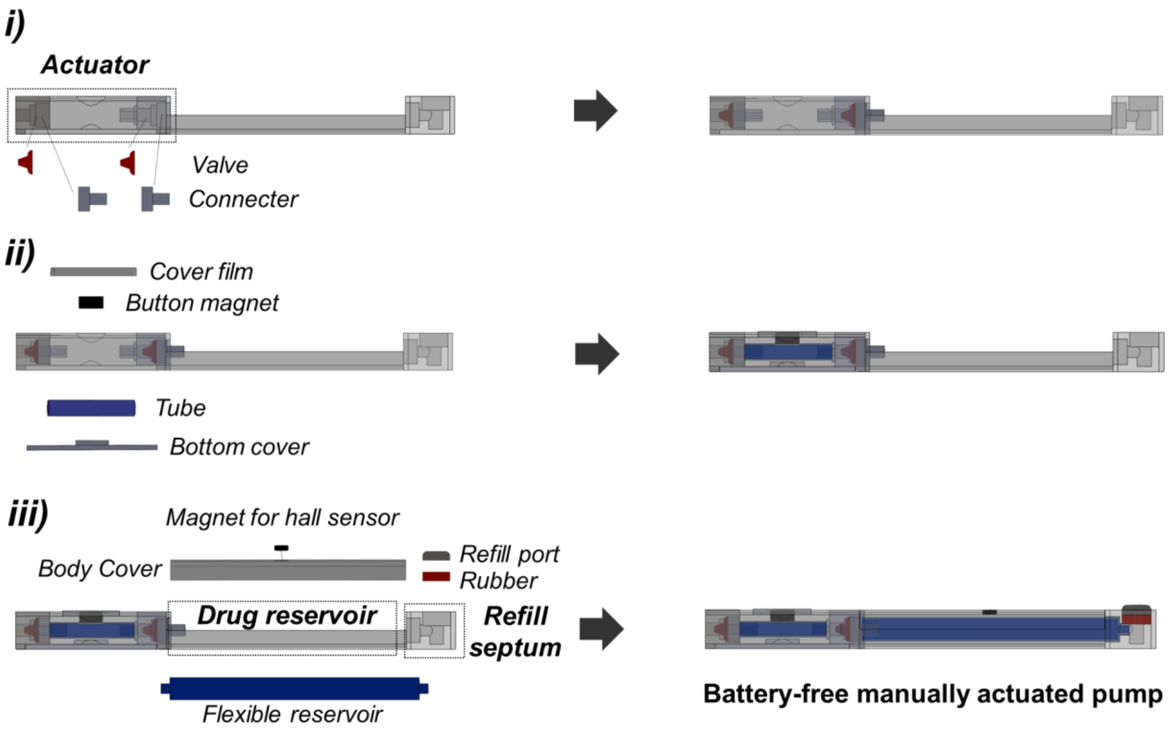

Fig. S1. A detailed description of the battery-free manually actuated pump assembly procedures. The pump structure was designed in SolidWorks 2019, and each component was fabricated with VeroClear rigid plastic with a glossy finish using a 3D printer (Object30 Pro, Stratasys). An actuator was assembled with two check valves (DU015.001-154.01 rev.:01, Minivalve), a silicone tube (inner diameter 1 mm, outer diameter: 1.5 mm), and a button magnet (diameter: 2.5 mm; thickness: 1.6 mm). Both ends were covered with a cover film (50 µm thick polyurethane film) and a bottom cover, respectively. A drug reservoir was connected with a flexible reservoir made of a 50 µm thick polyurethane film and covered with a body cover with a built-in magnet (diameter: 1.5 mm; thickness: 0.8 mm) for the hall sensor. A refill septum was made of rubber (diameter: 3 mm) and a refill port. All constituent components were assembled and bonded using a medical epoxy (Epo-Tek 301, Epoxy Technology).

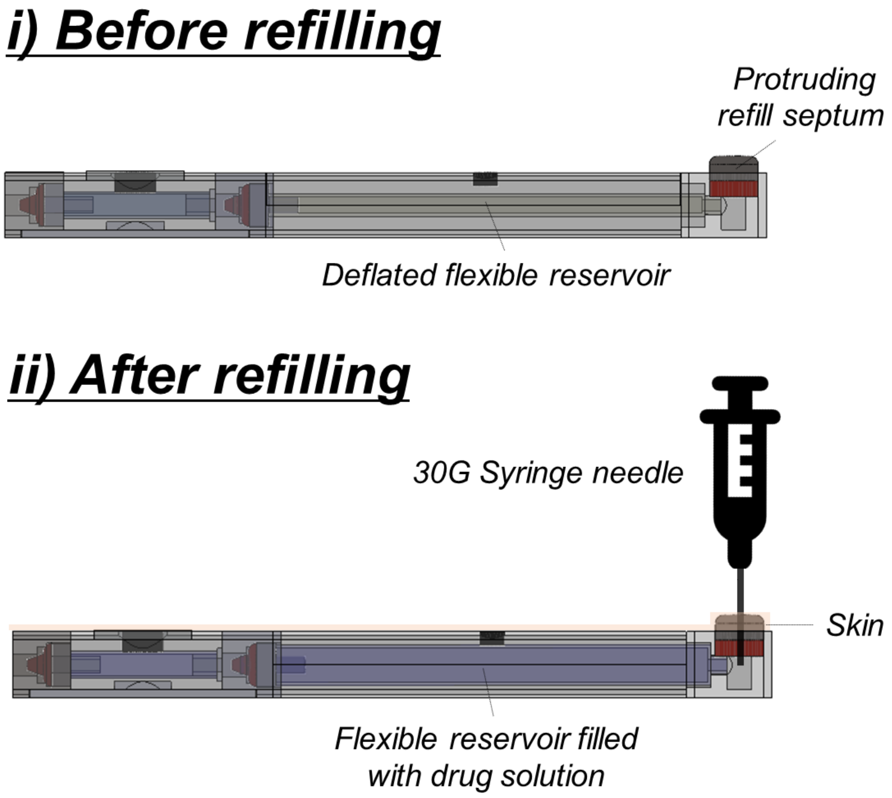

Fig. S2. Refilling procedure of the battery-free manually actuated pump

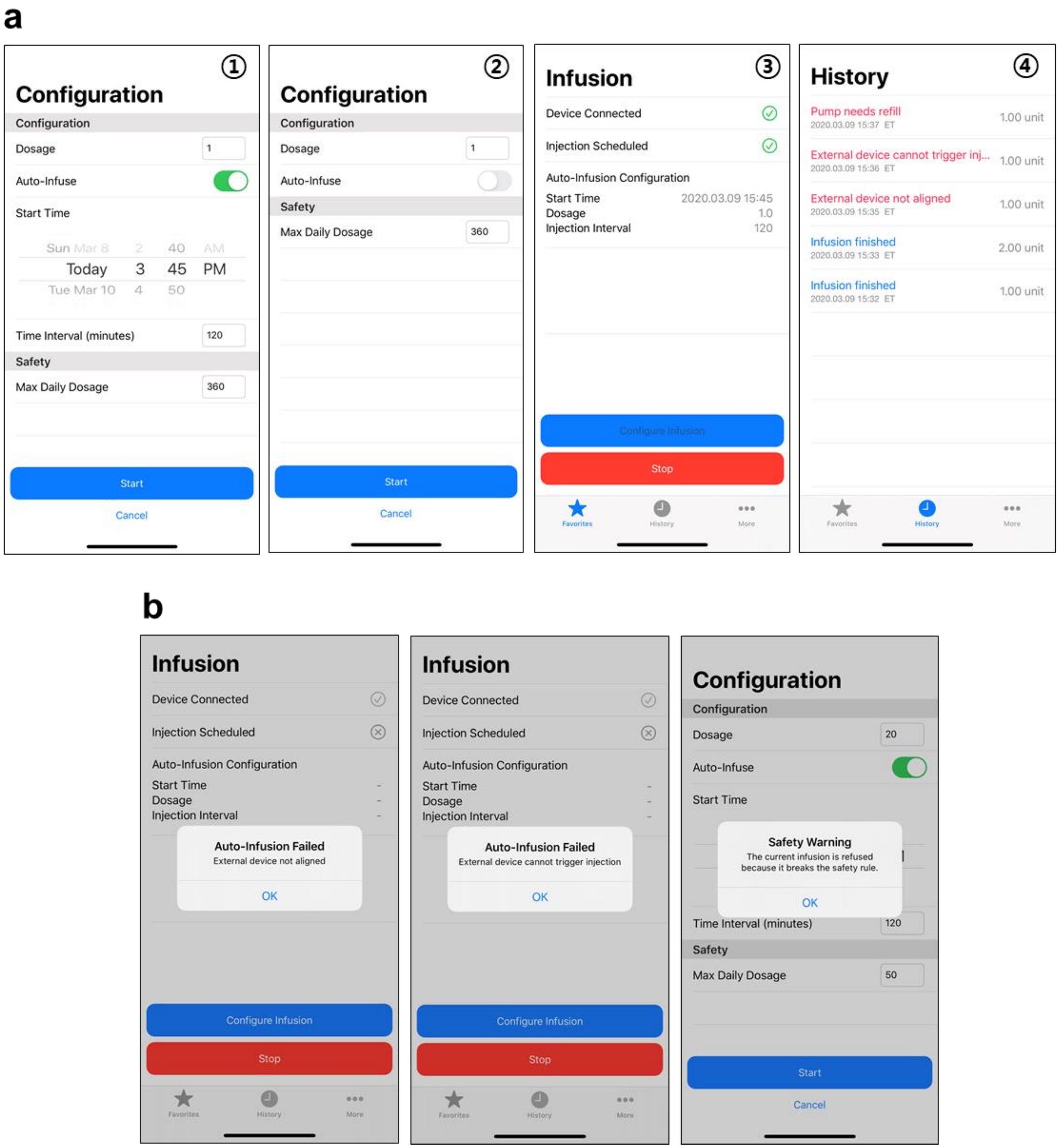

a
①
Configuration
Configuration
Dosage
1
Auto-Infuse
Start Time
Sun Mar 8 2 40 AM
Today 3 45 PM
Tue Mar 10 4 50
Time Interval (minutes)
120
Safety
Max Daily Dosage
360
Start
Cancel
②
Configuration
Configuration
Dosage
1
Auto-Infuse
Safety
Max Daily Dosage
360
Start
Cancel
③
Infusion
Device Connected
Injection Scheduled
Auto-Infusion Configuration
Start Time 2020.03.09 15:45
Dosage 1.0
Injection Interval 120
Configure Infusion
Stop
Favorites
History
More
④
History
Pump needs refill 1.00 unit
2020.03.09 15:37 ET
External device cannot trigger inj... 1.00 unit
2020.03.09 15:36 ET
External device not aligned 1.00 unit
2020.03.09 15:35 ET
Infusion finished 2.00 unit
2020.03.09 15:33 ET
Infusion finished 1.00 unit
2020.03.09 15:32 ET
Favorites
History
More
b
Infusion
Device Connected
Injection Scheduled
Auto-Infusion Configuration
Start Time -
Dosage -
Injection Interval -
Auto-Infusion Failed
External device not aligned
OK
Configure Infusion
Stop
Favorites
History
More
Infusion
Device Connected
Injection Scheduled
Auto-Infusion Configuration
Start Time -
Dosage -
Injection Interval -
Auto-Infusion Failed
External device cannot trigger injection
OK
Configure Infusion
Stop
Favorites
History
More
Configuration
Configuration
Dosage
20
Auto-Infuse
Start Time
Safety Warning
The current infusion is refused
because it breaks the safety rule.
OK
Time Interval (minutes)
120
Safety
Max Daily Dosage
50
Start
Cancel

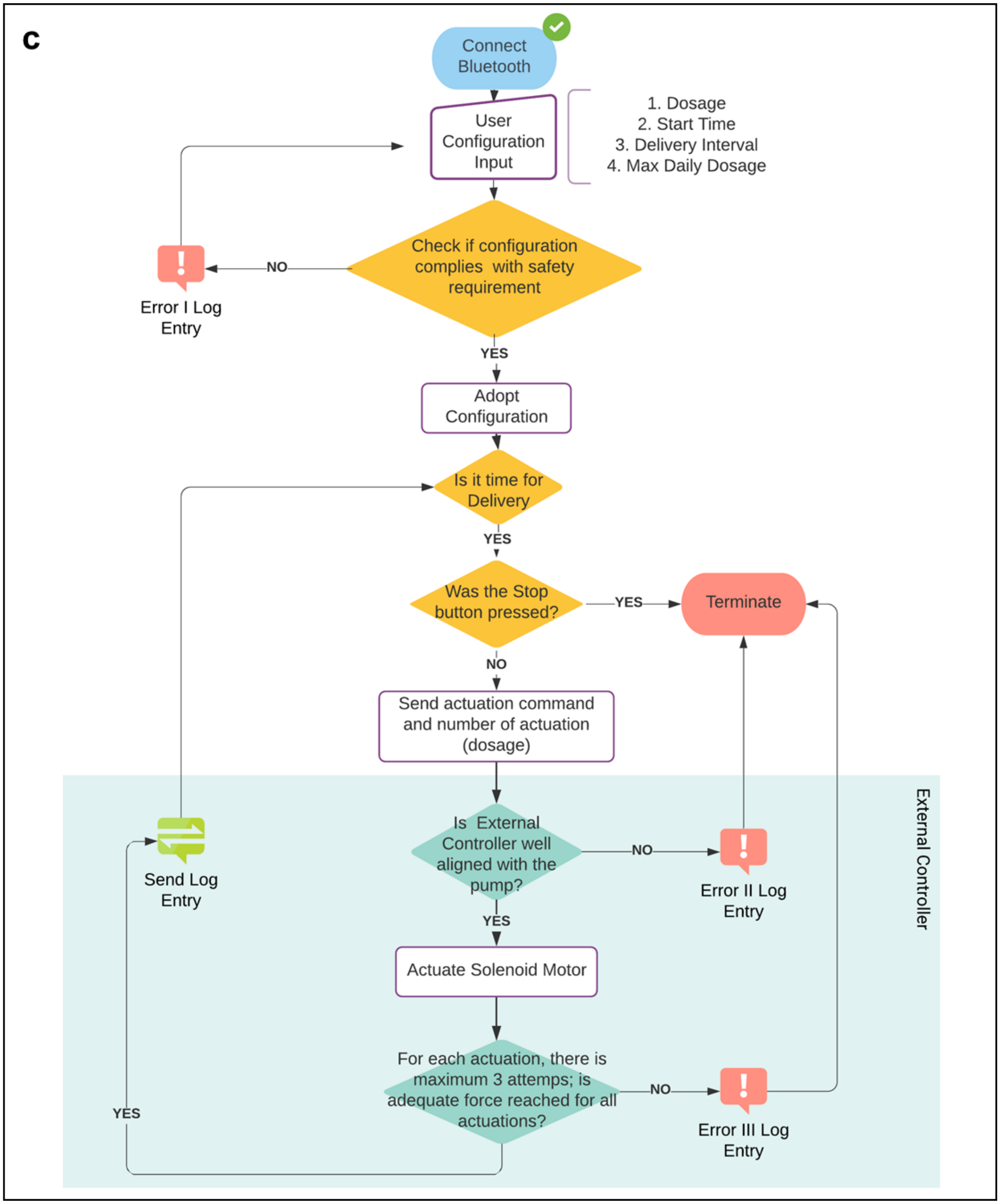

Fig. S3. Logic flowchart for the mobile app program Photo credit: Canchen Li, Massachusetts Institute of Technology.

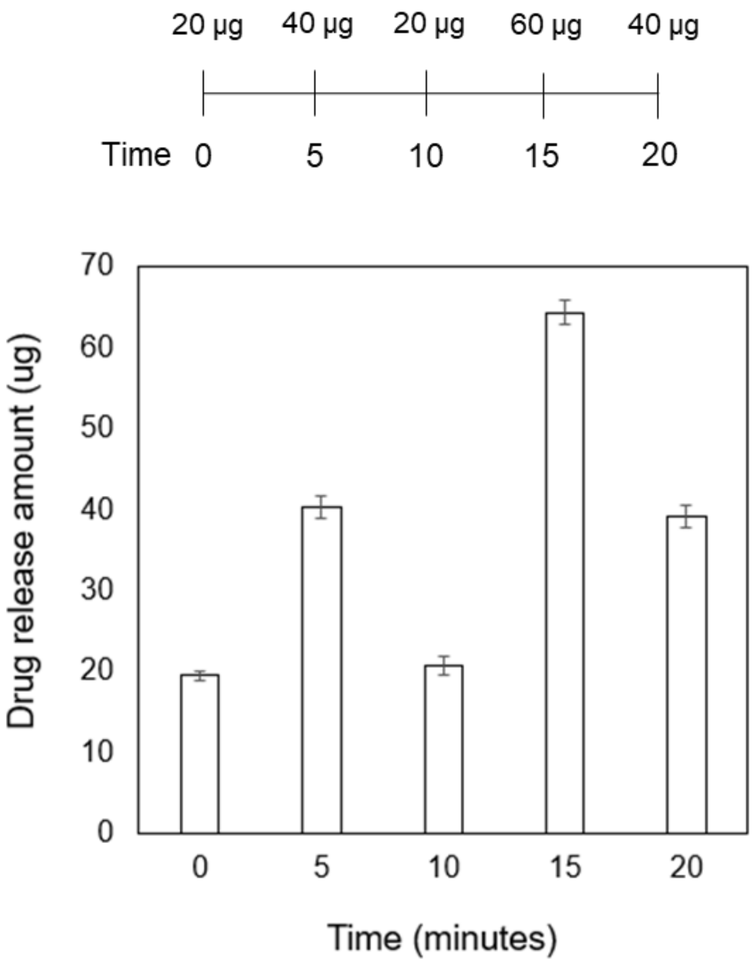

Fig. S4. For programmable control evaluation, we first programmed the device to infuse in sequence 20, 40, 20, 60, and 40 μg of the drug while setting the drug infusion interval to 5 minutes and the maximum permissible daily infusion amount to 200 μg. Consequently, the infusion of the drug was successfully carried out at the scheduled time points. Here, the pump was designed to infuse 20 μg per actuation. The error bars are the s.d.

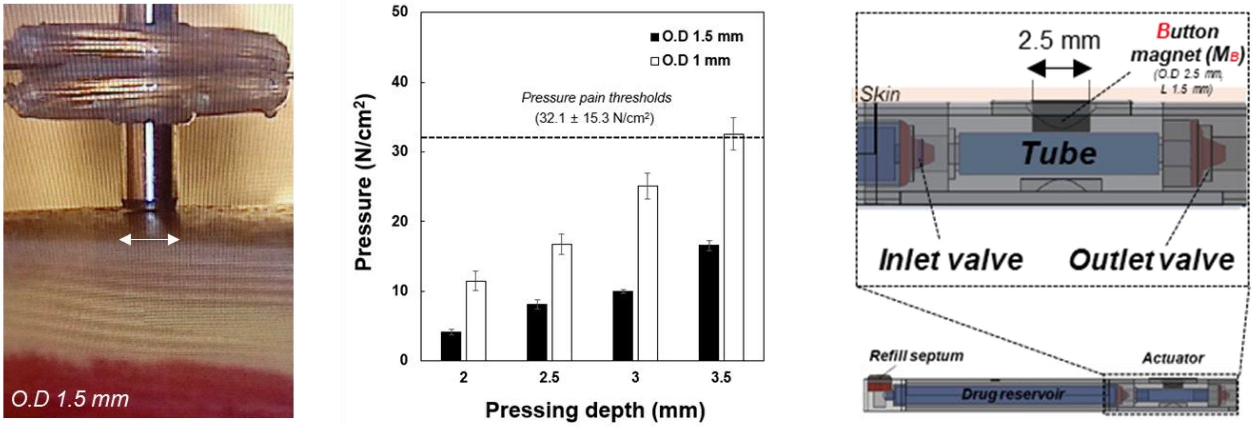

Fig. S5. Triggering characterization – inadvertent triggering mitigation

Two types of actuating rods (outer diameters: 1 and 1.5 mm) were attached to a mechanical testing machine (Instron 5942 Series Universal Testing System). In separate experiments, each was pressed down perpendicularly at a constant speed (1 mm/s) against the surface of the synthetic human tissues (SynTissue®) with a maximum depth of 3.5 mm. The synthetic skin was fully immersed in distilled water at room temperature to keep it in a hydrated state until testing. All experiments were performed in triplicate for each type of actuating rod. Here, the button magnet in the actuator is designed to be 2.5 mm to prevent an accidental or unwanted actuation and confirmed that the pump was not driven when pressed with actuating rod over 2.5 mm, which is much smaller than a baby's small finger (> 9 mm) *(43)*. Photo credit: Seungho Lee, Massachusetts Institute of Technology

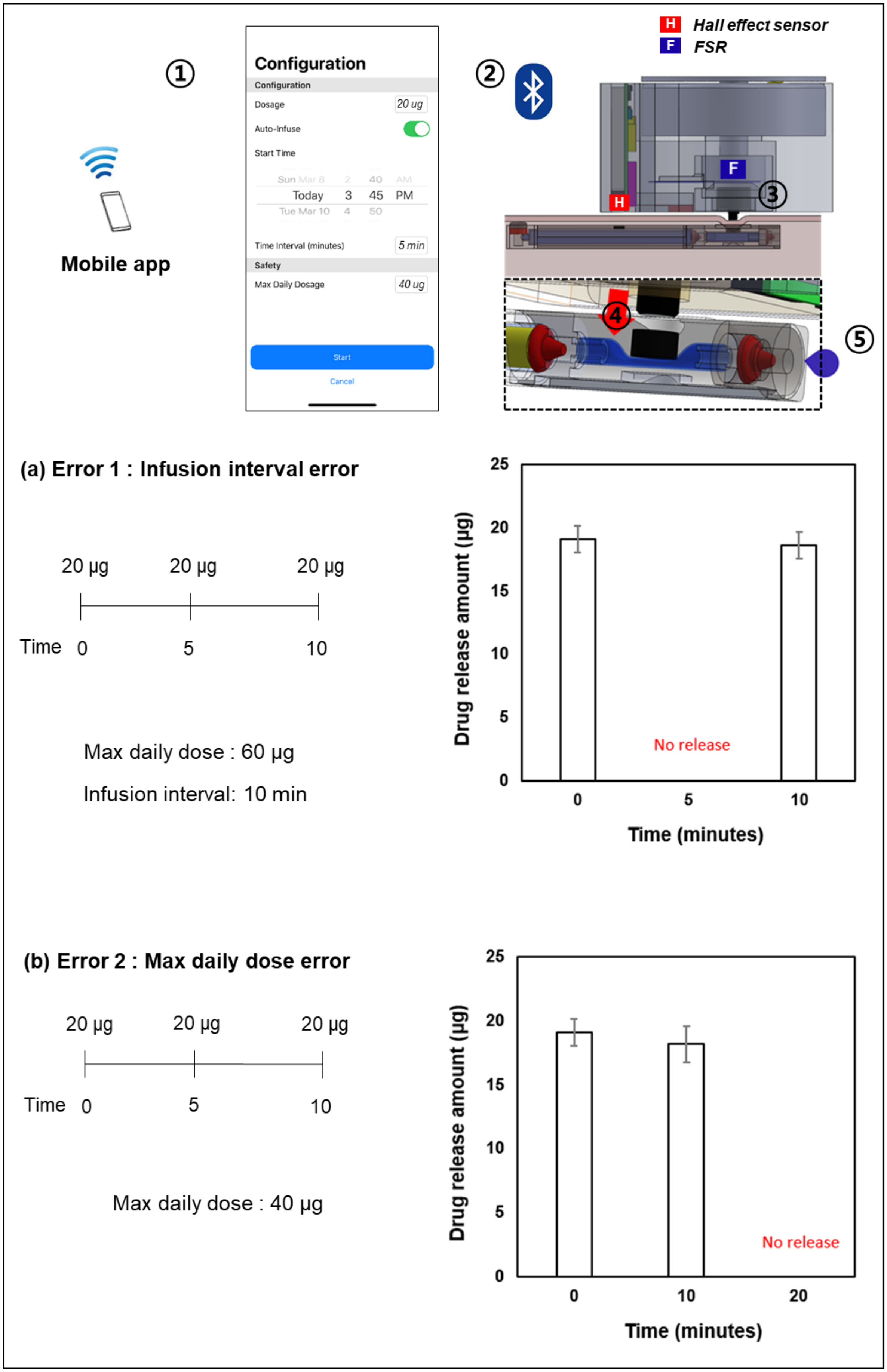

Hall effect sensor
FSR
①
Mobile app
Configuration
Configuration
Dosage
20 ug
Auto-Infuse
Start Time
Sun Mar 8 2 40 AM
Today 3 45 PM
Tue Mar 10 4 50
Time Interval (minutes)
5 min
Safety
Max Daily Dosage
40 ug
Start
Cancel
②
③
④
⑤
(a) Error 1 : Infusion interval error
20 µg 20 µg 20 µg
Time 0 5 10
Max daily dose : 60 µg
Infusion interval: 10 min
Drug release amount (µg)
Time (minutes)
No release
(b) Error 2 : Max daily dose error
20 µg 20 µg 20 µg
Time 0 5 10
Max daily dose : 40 µg
Drug release amount (µg)
Time (minutes)
No release

Fig. S6. As a safety feature, the mobile app is programmed to preset limits on the drug dosage and dosing schedule to avoid overdose. For Error 1, violated minimum infusion interval: we intentionally set the allowable minimum infusion interval to 10 min and tried to infuse 20 μg (1 actuation) of the drug at 0, 5, and 10 min. As a result of the programming, it was observed that the drug was infused at 0 and 10 minutes. However, at 5 minutes, the interval between two consecutive orders (5 minutes) was less than 10 minutes, so there was no drug infusion. For Error 2, exceeded daily maximum infusion dose error: The maximum allowable daily dose was set to 40 μg. An infusion order for the 20 μg dose was continuously generated at 10-minute intervals without violating the preset Error1 scenario. As a result, the drug was infused in a total dose of 40 μg. However, in the third order, because the total dose set at 40 μg was exceeded, the drug was not infused. Here, the pump was designed to infuse 20 μg per actuation. The error bars are the s.d.

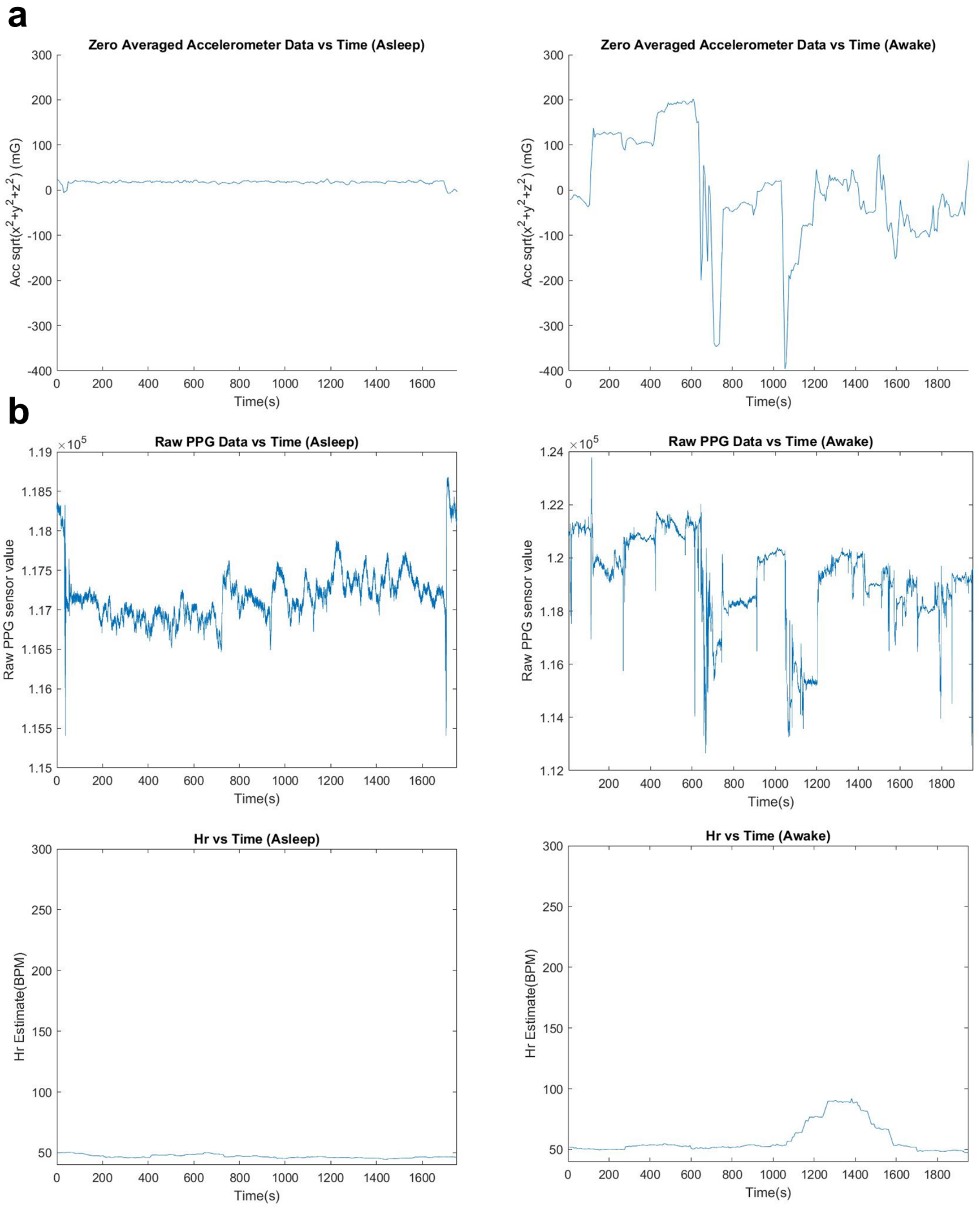
a
Zero Averaged Accelerometer Data vs Time (Asleep)
Zero Averaged Accelerometer Data vs Time (Awake)
Acc sqrt(x^2+y^2+z^2) (mG)
Time(s)
b
Raw PPG Data vs Time (Asleep)
Raw PPG Data vs Time (Awake)
Raw PPG sensor value
×10^5
Time(s)
Hr vs Time (Asleep)
Hr vs Time (Awake)
Hr Estimate(BPM)
Time(s)

Fig. S7. Sensors for daily rhythms in sleep and heart rate (a) Accelerometer: Accelerometer data vs. time for both the asleep and awake datasets. The raw accelerometer datasets are zero averaged then 5 points moving averagely filtered to generate these traces. (b) Photoplethysmography (PPG)**:** Heart rate data vs. time computed from the maximal spectral power of the filtered PPG signals for both the asleep and awake datasets. The FIR filter used is a bandpass filter constructed using MATLAB's signal processing toolbox implementation.

Movie S1 Video of a jet-like infusion

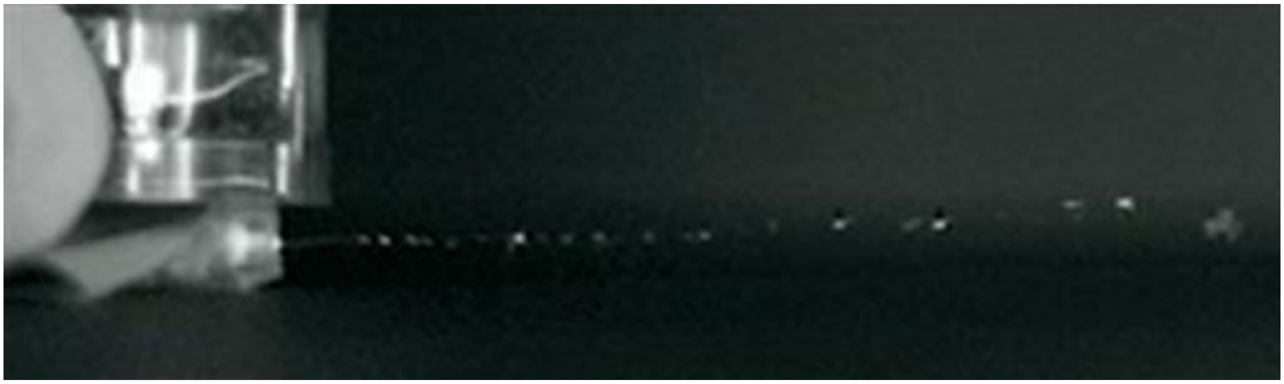

REFERENCES AND NOTES

1. K. Awad, M. C. Serban, P. Penson, D. P. Mikhailidis, P. P. Toth, S. R. Jones, M. Rizzo, G. Howard, G. Y. H. Lip, M. Banach; Lipid and Blood Pressure Meta-analysis Collaboration (LBPMC) Group, Effects of morning vs evening statin administration on lipid profile: A systematic review and meta-analysis. *J. Clin. Lipidol.* **11**, 972–985.e9 (2017).

2. B. L. Carter, E. A. Chrischilles, G. Rosenthal, B. M. Gryzlak, E. L. Eisenstein, M. W. Vander Weg, Efficacy and safety of nighttime dosing of antihypertensives: Review of the literature and design of a pragmatic clinical trial. *J. Clin. Hypertens.* **16**, 115–121 (2014).

3. J. M. Smyth, M. H. Soefer, A. Hurewitz, A. Kliment, A. A. Stone, Daily psychosocial factors predict levels and diurnal cycles of asthma symptomatology and peak flow. *J. Behav. Med.* **22**, 179–193 (1999).

4. M. C. Cohen, K. M. Rohtla, C. E. Lavery, J. E. Muller, M. A. Mittleman, Meta-analysis of the morning excess of acute myocardial infarction and sudden cardiac death. *Am. J. Cardiol.* **79**, 1512–1516 (1997).

5. V. Petrenko, C. Dibner, Circadian orchestration of insulin and glucagon release. *Cell Cycle* **16**, 1141–1142 (2017).

6. M. Riedel, B. Hoeft, W. F. Blum, A. von zur Mühlen, G. Brabant, Pulsatile growth hormone secretion in normal-weight and obese men: Differential metabolic regulation during energy restriction. *Metabolism* **44**, 605–610 (1995).

7. S. Marchisello, A. Di Pino, R. Scicali, F. Urbano, S. Piro, F. Purrello, A. M. Rabuazzo, Pathophysiological, molecular and therapeutic issues of nonalcoholic fatty liver disease: An overview. *Int. J. Mol. Sci.* **20**, 1948 (2019).

8. G. Kaur, C. Phillips, K. Wong, B. Saini, Timing is important in medication administration: A timely review of chronotherapy research. *Int. J. Clin. Pharm.* **35**, 344–358 (2013).

9. N. Ozturk, D. Ozturk, Z. Pala-Kara, E. Kaptan, S. Sancar-Bas, N. Ozsoy, S. Cinar, G. Deniz, X. M. Li, S. Giacchetti, F. Lévi, A. Okyar, The immune system as a chronotoxicity target of the anticancer mTOR inhibitor everolimus. *Chronobiol. Int.* **35**, 705–718 (2018).

10. M. Kraft, R. J. Martin, Chronobiology and chronotherapy in medicine. *Dis. Mon.* **41**, 506–575 (1995).

11. E. Callaway, H. Ledford, Medicine Nobel awarded for work on circadian clocks. *Nature***550**, 18 (2017).

12. M. T. Brown, J. K. Bussell, Medication adherence: WHO cares?*Mayo Clin. Proc.* **86**, 304–314 (2011).

13. R. R. Henry, J. Rosenstock, D. K. Logan, T. R. Alessi, K. Luskey, M. A. Baron, Randomized trial of continuous subcutaneous delivery of exenatide by ITCA 650 versus twice-daily exenatide injections in metformin-treated type 2 diabetes. *Diabetes Care* **36**, 2559–2565 (2013).

14. S. Palomba, A. Falbo, A. Di Cello, C. Materazzo, F. Zullo, Nexplanon: The new implant for long-term contraception. A comprehensive descriptive review. *Gynecol. Endocrinol.* **28**, 710–721 (2012).

15. D. S. Kohane, Microparticles and nanoparticles for drug delivery. *Biotechnol. Bioeng.* **96**, 203–209 (2007).

16. B. A. Yentzer, F. T. Camacho, T. Young, J. M. Fountain, A. R. Clark, S. R. Feldman, Good adherence and early efficacy using desonide hydrogel for atopic dermatitis: Results from a program addressing patient compliance. *J. Drugs Dermatol.* **9**, 324–329 (2010).

17. J. Li, D. J. Mooney, Designing hydrogels for controlled drug delivery. *Nat. Rev. Mater.* **1**, 16071 (2016).

18. M. R. Prausnitz, R. Langer, Transdermal drug delivery. *Nat. Biotechnol.* **26**, 1261–1268 (2008).

19. Y. C. Kim, J. H. Park, M. R. Prausnitz, Microneedles for drug and vaccine delivery. *Adv. Drug Deliv. Rev.* **64**, 1547–1568 (2012).

20. L. W. Kleiner, J. C. Wright, Y. Wang, Evolution of implantable and insertable drug delivery systems. *J. Control. Release* **181**, 1–10 (2014).

21. R. Farra, N. F. Sheppard, L. McCabe, R. M. Neer, J. M. Anderson, J. T. Santini, M. J. Cima, R. Langer, First-in-human testing of a wirelessly controlled drug delivery microchip. *Sci. Transl. Med.* **4**, 122ra21 (2012).

22. S. Belverud, A. Mogilner, M. Schulder, Intrathecal pumps. *Neurotherapeutics* **5**, 114, 122 (2008).

23. N. Mikhail, Quick-release bromocriptine for treatment of type 2 diabetes. *Curr. Drug Deliv.* **8**, 511–516 (2011).

24. J. E. Heeg, P. E. De Jong, G. K. Van der Hem, D. De Zeeuw, Efficacy and variability of the antiproteinuric effect of ACE inhibition by lisinopril. *Kidney Int.***36**, 272–279 (1989).

25. H. Pijl, S. Ohashi, M. Matsuda, Y. Miyazaki, A. Mahankali, V. Kumar, R. Pipek, P. Iozzo, J. L. Lancaster, A. H. Cincotta, R. A. DeFronzo, Bromocriptine: A novel approach to the treatment of type 2 diabetes. *Diabetes Care* **23**, 1154–1161 (2000).

26. A. H. Cincotta, T. A. MacEachern, A. H. Meier, Bromocriptine redirects metabolism and prevents seasonal onset of obese hyperinsulinemic state in Syrian hamsters. *Am. J. Physiol.* **264**, E285–E293 (1993).

27. R. A. DeFronzo, Bromocriptine: A sympatholytic, D2-dopamine agonist for the treatment of type 2 diabetes. *Diabetes Care* **34**, 789–794 (2011).

28. F. P. Pons-Faudoa, A. Ballerini, J. Sakamoto, A. Grattoni, Advanced implantable drug delivery technologies: Transforming the clinical landscape of therapeutics for chronic diseases. *Biomed. Microdevices* **21**, 47 (2019).

29. H. Joo, Y. Lee, J. Kim, J. S. Yoo, S. Yoo, S. Kim, A. K. Arya, S. Kim, S. H. Choi, N. Lu, H. S. Lee, S. Kim, S. T. Lee, D. H. Kim, Soft implantable drug delivery device integrated wirelessly with wearable devices to treat fatal seizures, *Sci. Adv.* **7**, eabd4639 (2021).

30. J. Koo, S. B. Kim, Y. S. Choi, Z. Xie, A. J. Bandodkar, J. Khalifeh, Y. Yan, H. Kim, M. K. Pezhouh, K. Doty, G. Lee, Y. Y. Chen, S. M. Lee, D. D'Andrea, K. Jung, K. H. Lee, K. Li, S. Jo, H. Wang, J. H. Kim, J. Kim, S. G. Choi, W. J. Jang, Y. S. Oh, I. Park, S. S. Kwak, J. H. Park, D. Hong, X. Feng, C. H. Lee, A. Banks, C. Leal, H. M. Lee, Y. Huang, C. K. Franz, W. Z. Ray, M. MacEwan, S. K. Kang, J. A. Rogers, Wirelessly controlled, bioresorbable drug delivery device with active valves that exploit electrochemically triggered crevice corrosion. *Sci. Adv.* **6**, eabb1093 (2020).

31. K. W. Fels, M. S. Cunha, G. P. Sturtz, R. Gemperli, M. C. Ferreira, Evaluation of cutaneous abdominal wall sensibility after abdominoplasty. *Aesthetic Plast. Surg.* **29**, 78–82 (2005).

32. I. Catambing, M. Villa, Ultrasonographic measurement of skin and subcutaneous thickness at insulin injection sites among adult Filipinos with diabetes, *J. ASEAN Fed. Endocr. Soc.* **29**, 24–32 (2014).

33. Adult skin. SynDaver, Accessed July 25, 2020; https://syndaver.com/product/adult-skin/.

34. U. Sharma, D. Concagh, L. Core, Y. Kuang, C. You, Q. Pham, G. Zugates, R. Busold, S. Webber, J. Merlo, R. Langer, G. M. Whitesides, M. Palasis, The development of bioresorbable composite polymeric implants with high mechanical strength. *Nat. Mater.* **17**, 96–103 (2018).

35. D. Deiss, A. Szadkowska, D. Gordon, A. Mallipedhi, I. Schütz-Fuhrmann, E. Aguilera, C. Ringsell, C. De Block, C. Irace, Clinical practice recommendations on the routine use of eversense, the first long-term implantable continuous glucose monitoring system. *Diabetes Technol. Ther.* **21**, 254–264 (2019).

36. G. Cappon, G. Acciaroli, M. Vettoretti, A. Facchinetti, G. Sparacino, Wearable continuous glucose monitoring sensors: A revolution in diabetes treatment. *Electronics* **6**, 65 (2017).

37. B. L. Smarr, D. C. Burnett, S. M. Mesri, K. S. J. Pister, L. J. Kriegsfeld, A wearable sensor system with circadian rhythm stability estimation for prototyping biomedical studies. *IEEE Trans. Affect. Comput.* **7**, 220–230 (2016).

38. D. Adam, Core concept: Emerging science of chronotherapy offers big opportunities to optimize drug delivery, *Proc. Natl. Acad. Sci. U.S.A.* **116**, 21957–21959 (2019).

39. T. Tan, S. W. Watts, R. P. Davis, Drug delivery: Enabling technology for drug discovery and development. iPRECIO Micro Infusion Pump: Programmable, refillable, and implantable. **2**, 44 (2011).

40. S. Ho, S. Hee, Y. Chan, J. Hoon, M. Na, C. Rim, C. Ho, B. Hwi, C. Lee, Y. Min, Y. Bin, Magnetically-driven implantable pump for on-demand bolus infusion of short-acting glucagon-like peptide-1 receptor agonist, *J. Control. Release* **325**, 111–120 (2020).

41. R. Farid, K. Binz, J. A. Emerson, F. Murdock, Accuracy and precision of the SynchroMed II pump. *Neuromodulation* **22**, 805–810 (2019).

42. R. Elfiyani, A. Amalia, S. Y. Pratama, Effect of using the combination of tween 80 and ethanol on the forming and physical stability of microemulsion of eucalyptus oil as antibacterial. *J. Young Pharm.* **9**, s1–s4 (2017).

43. B. Hohendorff, C. Weidermann, K. J. Burkhart, P. M. Rommens, K. J. Prommersberger, M. A. Konerding, Lengths, girths, and diameters of children's fingers from 3 to 10 years of age. *Ann. Anat.* **192**, 156–161 (2010).
